# LIVE-streaming 3D images: A neuroscience approach to full-body illusions

**DOI:** 10.3758/s13428-021-01659-6

**Published:** 2021-09-28

**Authors:** D. M. L. de Boer, F. Namdar, M. Lambers, A. Cleeremans

**Affiliations:** 1 School of Psychology and Counselling, Faculty of Health, Queensland University of Technology, Kelvin Grove, Queensland 4059 Australia; 2 Institute of Health and Biomedical Innovation, Queensland University of Technology, Brisbane, Australia; 3 Design doc, Ghent Office, Woodrow Wilsonplein 9, 9000 Ghent, Belgium; 4 Institute for Vision and Graphics, University of Siegen, Siegen, Germany; 5 Consciousness, Cognition, and Computation Group (CO3), Centre for Research in Cognition and Neurosciences (CRCN), ULB Neuroscience Institute (UNI), Université libre de Bruxelles (ULB), Avenue F.D. Roosevelt 50, CP191, 1050 Brussels, Belgium

**Keywords:** 3D, Streaming, Augmented reality (AG), Virtual reality (VR), Game technology, Gaming, Full-body illusion (FBI), Out-of-body experience (OBE), Brain stimulation, Neuroscience methods, Rehabilitation

## Abstract

Inspired by recent technological advances in the gaming industry, we used capture cards to create and LIVE-stream high quality 3D-images. With this novel technique, we developed a real-life stereoscopic 3D full-body illusion paradigm (3D projection). Unlike previous versions of the full-body illusion that rely upon unwieldy head-mounted displays, this paradigm enables the unobstructed investigation of such illusions with neuroscience methods (e.g., transcranial direct current stimulation, transcranial magnetic stimulation, electroencephalography, and near-infrared spectroscopy) and examination of their neural underpinnings. This paper has three aims: (i) to provide a step-by-step guide on how to implement 3D LIVE-streaming, (ii) to explain how this can be used to create a full-body illusion paradigm; and (iii) to present evidence that documents the effectiveness of our methods (de Boer et al., 2020), including suggestions for potential applications. Particularly significant is the fact that 3D LIVE-streaming is not GPU-intensive and can easily be applied to any device or screen that can display 3D images (e.g., TV, tablet, mobile phone). Therefore, these methods also have potential future clinical and commercial benefits. 3D LIVE-streaming could be used to enhance future clinical observations or educational tools, or potentially guide medical interventions with real-time high-quality 3D images. Alternatively, our methods can be used in future rehabilitation programs to aid recovery from nervous system injury (e.g., spinal cord injury, brain damage, limb loss) or in therapies aimed at alleviating psychosis symptoms. Finally, 3D LIVE-streaming could set a new standard for immersive online gaming as well as augmenting online and mobile experiences (e.g., video chat, social sharing/events).

**Supplementary Information:**

The online version contains supplementary material available at 10.3758/s13428-021-01659-6.

## Introduction

Out-of-body experiences (OBEs) are brief subjective episodes in which a person experiences the world from an illusory location outside their physical body (i.e., disembodiment). People typically report themselves floating or being able to observe themselves from above (i.e., autoscopy). Despite their often vivid nature, OBEs are quite rare and usually short-lived. About one in ten people encounter such an experience at some point in their lifetime ( (Blackmore, 1982; Green, 1968; Irwin, 1985; Thonnard et al., 2008)). Importantly, OBEs offer a unique insight into the functional brain and, more precisely, how the mind and body work together to construct a coherent sense of self in space and time ((Metzinger, 2013); for a critical review (Aspell et al., 2012)). However, because of their unpredictable nature, it has been difficult to study these experiences in a clinical setting (see (Blanke & Mohr, 2005) for a meta-analysis on autoscopic phenomena).

**Fig. 1:**
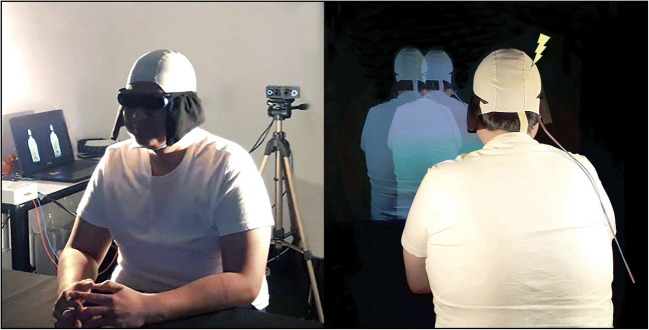
Stereoscopic 3D full-body illusion combined with HD-tDCS. Front (L) and back (R) view of full-body illusion paradigm based on LIVE-streaming stereoscopic 3D images with Bino 3D Player ( (Lambers, 2012)), here combined with high-definition transcranial direct current stimulation (HD-tDCS). The DC Stimulator Plus (neuroConn) can be seen to the side of the participant fastened on top of a table. Anode (red) vs. cathode (blue) electrode cables run down from the stimulator box up towards participant’s back. Electrodes (1–2 mm thick) are held into position on the scalp with electroconductive paste (Ten20, Weaver). An EEG cap is placed on top to ensure consistent adhesion to the scalp. See Fig. 4 for the electrode montage when the cap is removed

Recent technological advances have claimed to turn the tide. With the help of virtual reality techniques (VR), innovative paradigms have been created that enabled, for the first time, the systematic investigation of OBEs in the healthy population ( (Ehrsson, 2007; Lenggenhager et al., 2007)). These “full-body illusion” paradigms manipulate the experience of body ownership, which is the sense that our body (and its parts) is our own ( (Giummarra et al., 2008); also referred to as self-identification).^1^ In a typical full-body illusion paradigm, people observe a virtual body located a few feet in front of them through a head-mounted display (HMD). Whilst keeping still, for a few minutes they observe in front of them what they in real time feel happening to them (e.g., back-stroking). This illusory setting leads people to identify with the virtual body and to judge their spatial location to be closer to the external body than their actual location ( (Ehrsson, 2007; Lenggenhager et al., 2007)). However, because of practical, technical, and safety concerns (see Section 3), it has been challenging to combine neuroscience techniques (e.g., magnetic resonance imaging [MRI], electroencephalography [EEG], transcranial magnetic stimulation [TMS]) with the use of HMDs. As a consequence of these issues, the neural mechanisms that underpin such illusions remain under-studied ((Dieguez & Lopez, n.d.); e.g., (de Boer et al., 2020; Guterstam et al., 2015; Ionta et al., 2011)). Here, we address these challenges with the help of a novel technique: *3D LIVE-streaming*. This technique was used to create a stereoscopic 3D full-body illusion: video images are captured and streamed to a computer and are merged and displayed/projected in real time onto a large screen. Figure 1 shows the full setup combined with anodal transcranial direct current stimulation (tDCS). Unlike previous paradigms, this method gave us total freedom to investigate full-body illusions with neuroscience tools (e.g., tDCS, EEG, near-infrared spectroscopy [NIRS]).

The aim of this paper is to provide a step-by-step guide on how to create and integrate 3D LIVE-streaming into an experimental paradigm. The following sections will chronologically describe the different development stages, and will conclude with evidence supporting the effectiveness of these new methods (including a comparison to other approaches) and suggestions for future applications. First, the materials and streaming technique are explained in full detail. This includes the implementation steps taken to convert this into a full-body illusion paradigm. This section also covers details regarding the current constraints of combining noninvasive brain stimulation (NIBS) techniques with HMDs. Finally, the effectiveness of these new methods is illustrated using some data from our published experiment, which combined a full-body illusion paradigm with high definition tDCS (de Boer et al., 2020). Unlike previous paradigms, this allowed us to more precisely characterize the role of self-location (i.e., the experience of where *I* am in space, (Blanke, 2012)) and identify a neural mechanism that may underlie the breakdown in the unity between self and body.

## Developing 3D LIVE-streaming

### Game capture cards

Social media platforms like YouTube channels have rapidly changed the way we share information and communicate with each other on a global scale. Within the gaming community, there has been a rising trend to LIVE-stream one’s own game sessions online. Wider availability of high-speed internet and the advancement of game capture cards have played an important role in this new development. In recent years, game streaming platforms like Twitch (YouTube acquisition 2014) have become the go-to media through which gamers can watch and share their gaming experiences with the wider community. This has created a unique and thriving environment in which streamers and their viewers can interact with each other in ways hitherto impossible. However, most modern games are GPU [graphics processing unit]-intensive, meaning that a PC’s graphics processing unit can struggle to both render the games’ graphics and also encode them into the video footage necessary for streaming. This imposes restrictions on the streaming output it can generate. Similarly, other devices like game consoles (e.g., Nintendo Switch, PlayStation 4, Xbox One) currently offer limited-quality streaming. Game capture cards cleverly work around this problem by enabling audiovisual data to be streamed from one device to another without reducing the quality of the stream. Subsequently, (game) capture cards can be used to stream any audiovisual content at a professional quality (e.g., in full HD/4K resolution at 60 frames per second).

### From 2D to 3D LIVE-streaming

Inspired by these recent technological advancements, we decided to investigate whether game capture cards could be used to create a real-time stereoscopic 3D full-body illusion paradigm (see Introduction). Unlike previous paradigms that rely on using cumbersome HMDs, this would allow us to freely use neuroscience tools (e.g., tDCS, TMS, EEG, NIRS) to examine full-body illusions. Importantly, this streaming technique can be applied or extended to any screen that can display 3D images, be it large or small (e.g., a monitor, projector, tablet, mobile phone). Therefore, given some modifications, it can potentially benefit a variety of purposes, users, and settings (see Section 4). Until recently, LIVE-streaming 3D images had not been feasible. Until game capture cards came on the market, there had been no way to directly stream two separate video feeds to a computer without substantial loss in quality and frame drops. In addition, just a limited amount of software was developed to display 3D material (i.e., combined 2D images), let alone, software that would support real-time 3D streaming. However, with the use of identical capture cards, we hypothesized that two action cameras (i.e., one for each visual hemifield) could be directly linked to a computer via USB 3.0 ports. A specialized media player could then be used to encode and merge the two video feeds together to generate one 3D streamed output with minimal lag between the images. The next sections cover the three phases of this project: (i) the setup of the 3D camera rig; (ii) 3D LIVE-streaming with the Bino 3D Player; and (iii) the creation of a novel full-body illusion paradigm.

### Phase 1: Setup of the 3D camera rig

**Fig. 2:**
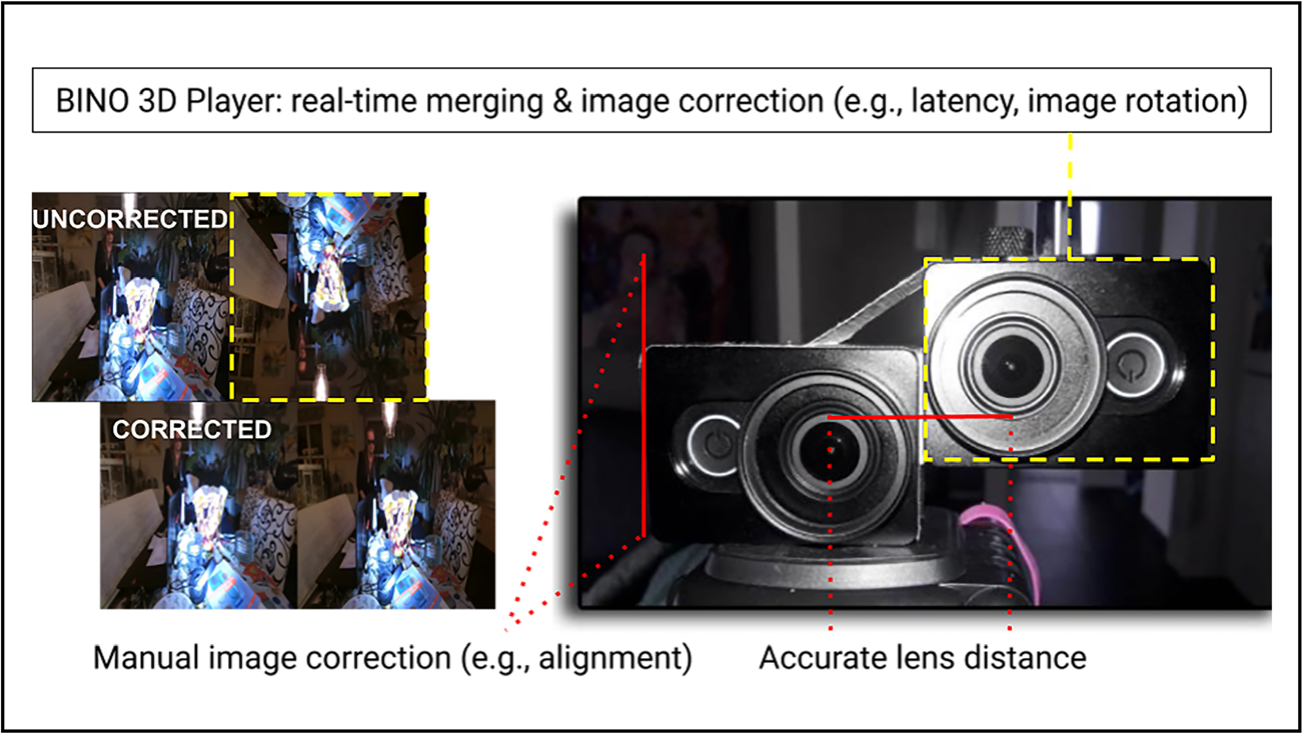
Stereoscopic 3D camera rig. Phase 1: Design of the stereoscopic 3D camera. Red markings: camera lenses had to be precisely (i) distanced and (ii) aligned in millimeters. Yellow markings: one camera had to be horizontally flipped (see “uncorrected” images). Phase 2: Bino 3D Player-software (1) synchronized, (2) aligned, (3) corrected, (4) prepared, and (5) presented the stereoscopic images in real-time (see “corrected” images, (Lambers, 2012)).

To capture stereoscopic 3D images, a camera rig equipped with two high-definition wide-angle action cameras (2 × 16 MP, F2.8 aperture, 155° ultra-wide-angle glass lens), each streaming at 60 frames per second, was constructed. A wide lens angle was necessary (i) in order for the lenses to capture enough light, and (ii) to create the illusion of more distance in the images (i.e., conditions that are generally lacking in laboratory settings). In addition, (iii) action cameras are relatively inexpensive options that offer high-quality image processing (in resolution, frames per second, etc.). To guarantee sharp, fluent, and easy-to-process 3D images, Full HD action cameras running at 60 frames per second were used (brand: Xiaomi Yi; 2K resolution). These cameras include a fisheye correction mode that limits the distortion on the edges of the images (i.e., wide-angle lenses tend to bend and deform the areas closer to the borders of the frames, making peripheral objects look bigger). Subsequently, some standard image corrections (e.g., movement stabilization, contrast corrections, white balance) had to be manually turned off to guarantee real-time image feedback and matching output of images (note: the cameras’ position did not move or change throughout the experiment). However, each camera lens is a unique product and may have slight variations between lenses (e.g., in lens angle, centre of focus, viewing range, etc.). Therefore, the supplier was contacted to make sure that the lenses were from the same production line besides having the same advertised specifications. The first task was to design a camera rig that would create proper 3D images. Importantly, the camera lenses needed to be precisely distanced and aligned with each other (with millimeter precision). Further, to guarantee a good 3D experience, a certain distance between the camera and objects was required (in meters, see Section 3). At this stage, LIVE-streaming and real-time tweaking of the relative camera positions was not yet possible. Therefore, the distances between the lenses were manually set and adjusted by one centimeter (7 cm, 6 cm, 5 cm, 4 cm, 3 cm) until an appropriate viewing angle was found. Such an angle would allow for effortless viewing of the 3D images without the need for too much eye-correction (using wide camera lenses). First, the two cameras were mounted on a stick, then short outdoor videos were shot under different light settings and lens distances (i.e., while walking or attached to the front of a bike). In the lab, the two separate videos were manually merged using DaVinci Resolve 15 editing software ( (Blackmagic Design Pty. Ltd, 2018)). The videos were synchronized through time codes and corrected for height differences between images. The border regions of the images that had no visible overlap were cropped out, and the resulting 3D image was zoomed in (this also removed the remaining distortion after applying the fisheye correction). Note that the latter steps lower the resolution of the 3D image. However, because the 3D image consisted of two 1080 p high-resolution images, this effect was negligible. A somewhat different approach was taken during the experimental phase (i.e., when a projector and screen were used instead of a large 3D TV; Phase 2). In short, once the correct settings had been identified with the right viewing angle, a specific casing for the montage could be made. This effectively meant that one of the cameras needed to be flipped horizontally for the lenses to be positioned close enough to create high-resolution stereoscopic 3D images (Fig. 2).

### Phase 2: 3D LIVE-streaming with Bino 3D player

Once the rig had been constructed, the cameras were connected to a PC through USB 3.0 ports. This was achieved by pairing each camera (via a micro HDMI to HDMI cable) with an identical AGPtek USB 3.0 capture card connected directly to the PC. Subsequently, the two separate video streams were merged in real time, creating one stereoscopic 3D LIVE image. The resulting image was projected onto a screen or wall. Throughout the experiment, the room was kept dark, and only the participant’s back was illuminated from each side (see Section 3, Fig. 5). Because the participant was sitting in the middle of the image, only the center of the images included visible content, and peripheral distortion was less of an issue (see Phase 1). The images were zoomed in and overprojected to the sides of the screen (i.e., onto black sheets). To stream and merge the images to a projector in real time, we used Bino 3D player software version 1.6.7 (Lambers, 2012) running on a Linux operating system (Ubuntu 18.04 LTS software with kernel 4.19.5).^2^ The following tasks were performed by the video player: (1) synchronization of input from two video streams, (2) adjustment of the vertical alignment of the streams, (3) correction of the horizontal orientation of one stream, (4) preparation of the video data for the stereoscopic screen, and (5) presentation of the resulting video data. The original stream synchronization code in Bino was targeted at video files with diverging frame timestamps, which requires synchronization for each frame. However, this does not work well when the two input streams come from capture devices (in the worst case, e.g., the streams take turns to wait for each other, resulting in frame loss). The synchronization of two streams from identical capture devices (task 1) was therefore implemented as a special case: only the first frame is synchronized to minimize stream differences, and for all following frames a constant frame rate of both devices is assumed. That way, the timing difference between the two streams is at most half of the frame duration (i.e., ~8.3 milliseconds for video streams with 60 frames per second). Furthermore, to avoid breaking immersion, the video processing tasks 2–4 had to be performed in a way that minimized latency between video frame capturing and display on screen. For this purpose, the Bino 3D player uses a GPU-based video processing pipeline that already automatically implemented tasks 3 and 4. Subsequently, task 2 was implemented as an additional vertical shift of texture coordinates during video frame merging, whereas the appropriate offset in pixels was determined during the calibration of the setup. In other words, while the latency (see above) is handled automatically by Bino, the alignment offsets between the two video streams had to be calibrated manually. For this purpose, a simple command script was created to prompt Bino to use the appropriate USB ports to synchronize the two video streams (task 1) and perform basic offset corrections (task 2). Having incorporated these steps, as of August 2018, the Bino 3D player-software was ready to LIVE-stream stereoscopic 3D images in full HD, running at 60 frames per second (Fig. S1).^3^

An important advantage of capturing images is that they are mere copies of the images being displayed on-screen or recorded through film. Therefore, a second GPU is not needed to (re) calculate those images, nor does the original computer have to do this all simultaneously (which would require a fast GPU). The system requirements for this technique are therefore moderate: to calculate the images without delay, only a mid-range graphics card is required (e.g., GeForce GTX 960/1060 and up), which has become more standard with the increased demand for accessing online video content (e.g., YouTube). To ensure that the streamed images are processed without frame drops or lag, we recommend a computer with a minimum of four cores (Intel i7, AMD Ryzen 7) and fast RAM (speed of 2400 MHz). For example, one camera capturing at 1080 p at 60 fps of uncompressed video data requires a data bandwidth of about 3000 Mbps. Using two cameras would normally double that amount, but because the cameras apply real-time data compression, the bandwidth size is kept within limits (note: although negligible upon visual inspection, data compression slightly reduces the streaming quality). To further decrease the bandwidth size, the audio streams of the cameras can be disabled. Section 3 describes how a novel full-body illusion paradigm was created by 3D LIVE-streaming. In addition, we present information regarding the current constraints of combining noninvasive brain stimulation (NIBS) techniques with HMDs.

## A novel 3D full-body illusion paradigm (3D projection)

**Fig. 3:**
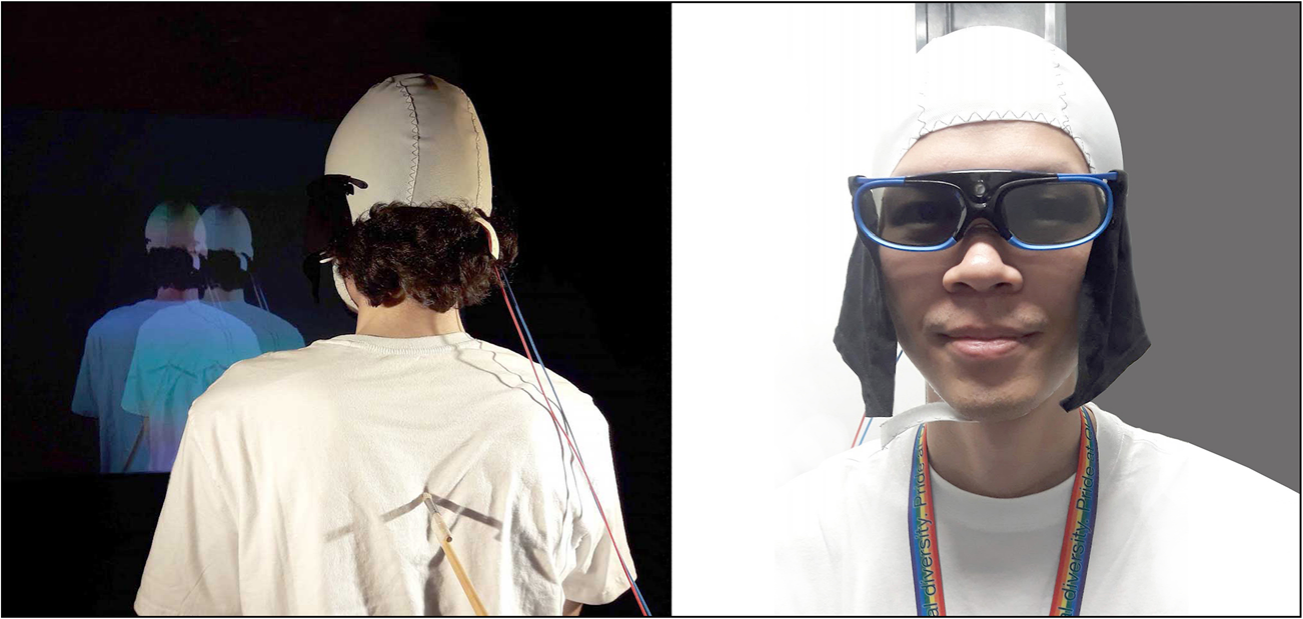
Procedure full-body illusion * high-definition tDCS. (L) Two cameras captured participants from behind, while they looked at themselves being stroked on the back projected in 3D in front of them. Stroking centered on the back (~20 cm length) at 50 strokes per minute. (R) The brain can be freely stimulated when using plastic 3D goggles to observe the illusion. Cotton cloths are attached to the goggles’ temples to prevent strong spotlights from hitting the lenses

Full-body illusions (FBIs) are typically simulated in a virtual environment using head-mounted displays (HMDs; (Ehrsson, 2007; Lenggenhager et al., 2007)). However, because of practical, technical, and safety concerns, it has been challenging to combine HMDs with neuroscience methods (e.g., MRI, EEG, TMS) and decipher the neural mechanisms that underpin such illusions (Dieguez & Lopez, n.d.; Ionta et al., 2011). This section will address some of the challenges that led us to develop 3D LIVE-streaming and create a novel FBI paradigm (i.e., a life-size 3D projection). The method is straightforward: high-quality images of two action cameras are captured and streamed to a computer and real-time merged and 3D projected onto a large screen (Fig. 3**)**. As can be seen, the video cameras LIVE-captured participants from behind, while they looked at themselves being stroked on the back projected life-size in 3D in front of them. This approach allows full access to the head and skull and can be easily combined with neuroscience tools (e.g., tDCS, TMS, EEG, and NIRS), here shown when high-definition tDCS is applied to the skull and plastic 3D goggles are used to observe the illusion.

### HMDs: Practical, technical, and safety concerns

**Fig. 4:**
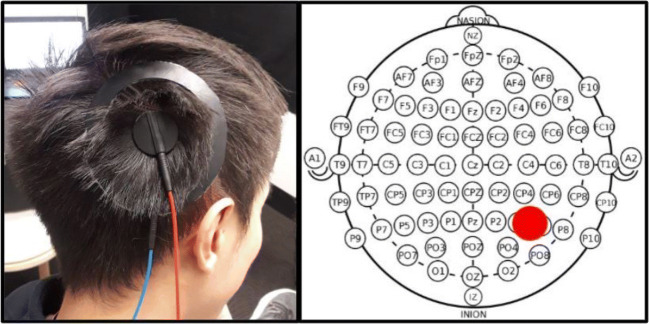
HD-tDCS montage and P4-P6 electrode positioning. (L) HD-tDCS red “anode” and blue “cathode” concentric center-ring montage to right angular gyrus;. (R) P4-P6 electrode positioning (Brodmann Area 39) in 10-10 International EEG system

HMDs are not easily combined with neuroscience methods that generally require free and unobstructed access to the head and skull. This section covers some of the challenges we faced when setting up our experiments and should not be regarded as a complete account. It mostly covers some careful considerations when combining noninvasive brain stimulation (NIBS) techniques with the use of HMDs. Considering tDCS, both conventional and newer electrode montages are not currently approved for use alongside tools or equipment that contain metal components. We used high-definition tDCS that constrains the current flow more specifically to the target regions (Fig. 4; (Bortoletto et al., 2016; Gbadeyan et al., 2016)). However, any metal in close proximity to the electrodes has the risk of heating or altering the current distribution (like also lesions do). For the same reasons, potential participants that have metal objects, scraps, or implants in or near the head, skull, or body should be screened out (Rossi et al., 2009; Rossi et al., 2011). This is a clear problem when using HMDs, but there are no such restrictions when combining 3D LIVE-streaming with NIBS techniques. Secondly, the space one has to work with is very limited when intermixing tDCS/tACS/tRNS with other equipment and they will often end up influencing each other (e.g., combined with EEG). Neuroscientists who frequently use these methods understand the difficulties involved when carefully setting up electrodes on the skull (which is a skill in its own right). Electrodes can be easily offset and/or their impedance can go down by having to put something large and heavy on the head as an HMD with cables hanging from it. This is also an issue when combining HMDs with other neuroscience tools (e.g., EEG and NIRS) and gets even more challenging when considering electrode positioning. Our target region was located just below the skull at the back corner of the head (i.e., where the band fixes the HMD to the head; Fig. 4). Subsequently, the HMD would partly rest on top of the electrodes, pulling on the montage and/or dislocating it over time (note: the rubber electrodes are held into place by a thin 1–2 mm layer of conductive gel, while the head cap offers just minimal protection). Our target region also made it difficult (even hazardous) to stand with full equipment on during the illusion: (i) the angular gyrus is a major cortical projection of the vestibular system important for balance and spatial orientation (Blanke, 2012); in addition, (ii) the standard electrode cables that connect to the stimulation box are just 1.5 meters long (e.g., reasons why participants sat in our experiment; Fig. 1). This gets even more problematic when participants are allowed to move around in simulation (e.g., (Swinkels et al., n.d.)).

Current technical and design constraints also limit the use of HMDs. HMDs that can render realistic, high-quality simulations still take up a lot of head space. These systems, mainly used for commercial gaming (e.g., the Oculus Rift, HTC Vive, or Valve Index), house relatively large and heavy displays. As such, an equally large and semi-rigid construction is needed to keep the HMD balanced and fixed onto the head (note: the weight of the display to the front needs to be somehow counterbalanced). In addition, the viewing angle must be kept stable over time; otherwise the images may not be observed well, the simulation might not work properly (i.e., the illusion in our case), risking all kinds of adverse events (e.g., eye-strain, headache, dizziness, nausea, motion sickness). Unfortunately, these are not trivial issues; adverse events and “simulation sickness” pose a serious problem for VR game developers, and make it a very unpleasant experience for users. A pilot study recently combined tDCS with VR as a therapy for post-traumatic stress ( (Wout-Frank et al., 2019)). They used The BraveMind visor, which has a slim, lightweight design that has the advantage of not obstructing the head too much or pulling it down. Consequently, simple polyester headbands can be used to strap the HMD onto the head (i.e., much like a visor hat). However, The BraveMind system (2003) does not contain a high-quality display that can render realistic 3D simulations comparable to new-generation systems.^4^ These high-tech devices carry motion sensors and fully integrated tracking systems, i.e., components that are not easily combined with NIBS techniques(LaValle, 2020).

Basically, until lightweight, high-quality HMD displays become commercially available, these systems are still quite fragile, expensive, and heavy devices made up of components that can neither be easily experimented with nor are they very suitable for routine use in clinical settings. As mentioned before, metal components embedded into the devices have the risk of heating up by any type of electrical current. Most high-tech equipment such as HMDs heats up in use, and there are strict design limits to how much temperature increase their cooling systems can handle before overheating. Unfortunately, until now, there has been insufficient data on what added effects nearby electrical stimulation has on technical devices located close to the head (e.g., preliminary results suggest that tDCS does not influence cardiac pacemaker function (Roncero et al., 2020)). Considering TMS or even tACS/tRNS, there are yet other reasons why intermixing this with HMDs should be avoided. The relatively strong magnetic pull of the TMS coil hampers the motion sensors, adding noise to the data signal. This will show as interference on the images, and its sensors could break down (e.g., accelerometer, gyroscope, magnetometer). Since these sensors are built to be highly sensitive to outside influences, a weak alternating current like tACS/tRNS could potentially pose a problem and hamper their functions.^5^ Such outcomes are not good for the device or for the participant (e.g., resulting in eye strain, headache, nausea, motion sickness). Cassani et al. (2020) recently reviewed studies that combined VR with NIBS techniques. Three of the 16 reviewed studies effectively combined a NIBS technique with the use of an HMD (Cassani et al., n.d.). The studies investigated potential novel therapies for post-traumatic stress (discussed pilot study (Wout-Frank et al., 2019); tDCS) and phobia (Deppermann et al., 2016; Notzon et al., 2015); TMS), with mixed results. Cassani et al. reported “[a current] lack of guidelines and best practices on how to combine VR and NIBS techniques” (abstract), since this *“*combination in therapeutic applications is recent*”* (p. 7). However, as noted above, the recency of such approaches might not have been the issue. Two of the reviewed studies used pre-VR stimulation protocols (i.e., TMS was not applied to the skull while participants wore an HMD) and none of them used an HMD that can render realistic (i.e., high-quality) 3D simulations (Cassani et al., n.d.). For the reasons stated above, pre-stimulation protocols currently offer the safest option to examine the potential benefits of VR therapy combined with brain stimulation. A full review unfortunately falls beyond the scope of this method paper.

In summary, there are practical, technical, and safety concerns when combining HMDs with neuroscience methods. The current-generation HMDs remain obstructive, cumbersome devices that are uncomfortable to wear and laborious to work with, especially for neuroscientists who require space and flexibility to experiment. Some HMD components are not safe when combined with other techniques that may hamper their function. Consequently, being part of a simulation and viewing images through an HMD (e.g., limited by a specific resolution, angle, field of view [FOV], frame rate) creates many variables that are out of the control of the researcher. Adverse events (e.g., eye strain, headache, dizziness, nausea, motion sickness) lie in wait and need to be carefully considered. 3D LIVE-streaming offers a good alternative to these problems by allowing researchers (and clinicians) free and unobstructed access to the head and skull. Furthermore, being able to observe real-life high-quality images means that 3D LIVE-streaming is the closest one can get to reality: it is not a simulation, and what can be captured does not need to be produced. The latter would consume more time and resources, and produce qualitatively inferior results.^6^ The details of the 3D LIVE-streamed full-body illusion paradigm are discussed next.

### A novel full-body illusion paradigm (3D LIVE-streamed)

**Fig. 5:**
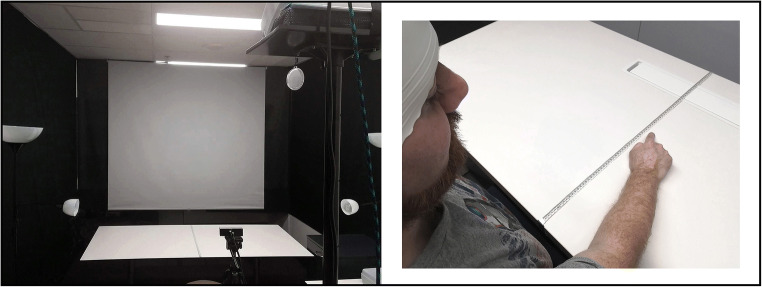
Setup 3D-HDtDCS lab. The participant sat on a stool behind a table located in the middle of the room (~200 cm behind the projection screen). The projector is seen ~400 cm in front of the screen behind the cameras. In front of the stool, a measuring tape is visible that counted out from the participant towards the screen (see right panel). On the measuring tape, participants indicated shifts in self-location or “proprioceptive drift” before and after the illusion (see (de Boer et al., 2020)). The room was kept dark except for two spotlights that illuminated the participant’s back from opposite directions

A novel full-body illusion (FBI) paradigm was developed with the use of 3D LIVE-streaming. Subsequently, several factors are important for the illusions to be effective: (i) the two video streams must be synchronized (Section 2, Phase 2); (ii) the participant should be aligned in terms of height, position (i.e., centered), and be approximately of the same size as the projected figure; and (iii) sufficient distance must be kept between objects (i.e., projector, cameras, participant, screen) for the 3D to work well, see Fig. 5. The technical equipment consisted of a set of two 3D-mounted action cameras (brand: Xiaomi Yi; 2K resolution) fastened on top of a tripod that was connected to an ASUS ROG Strix gaming laptop via two AGPtek USB 3.0 capture cards. In return, the gaming laptop is connected to an Optoma HD 3D projector via a high-speed HDMI cable. Note that a projector is not a requirement for this setup/3D LIVE-streaming to work. This was merely convenient to create a life-size illusion. In fact, the images displayed on a computer screen can be extended (copied) to any device that can display 3D images (e.g., a life-size 3D TV is a more costly alternative, see Section 2). We used a budget home theater projector with full-screen 3D projection capabilities (including two HDMI ports for direct input). Height and offset of the angle of the projection were checked each day and manually adjusted before participants came in (note: these options are readily available on the device and/or remote control). The Optoma HD 3D projector uses digital light projection with direct input (i.e., 8.4 ms response time at 120 Hz full HD; the room was fully air-conditioned). At 60 frames per second, it projected full HD stereoscopic 3D images onto a white screen positioned four meters away and two meters in front of the participant. To ensure a good quality projection, the room was kept dark except for two spotlights that illuminated participants’ backs from opposite directions (note: surroundings were blacked out; LED lights with different luminance were used to create more natural lighting). Importantly, this setup allowed participants to “observe” in front of them what they “felt” happening to them real time, creating the illusion of an out-of-body experience. Finally, to accommodate for the occasional tall or short participant, the cameras (and spotlights^7^) were placed respectively a few inches away from the participant and shifted upwards (tall participant) or towards the participant and shifted downwards (short participant). For this purpose, three premade sets of measurements were marked out on the ground and the tripod. This section will close by looking at some data demonstrating the effectiveness of this novel paradigm (see our published work (de Boer et al., 2020)).

With the use of 3D LIVE-streaming, we successfully combined a novel FBI-paradigm with high definition transcranial direct current stimulation (HD-tDCS). Unlike previous paradigms, this enabled us to more precisely characterize the role of self-location (i.e., the experience of where *I* am in space, *(*Blanke*,* 2012*)*) and identify a potential neural mechanism underlying self-identification (for details see (de Boer et al., 2020)). In our experiment we systematically manipulated the right angular gyrus (P4–P6; Brodmann Area 39), an area that is well known for its involvement in out-of-body experiences ((Blanke et al., 2002; De Ridder et al., 2007), Penfield in *(*Tong*,* 2003*)*). Figure 4 illustrates the electrode positioning on the scalp. The experiment included 36 healthy volunteers (24 females, 12 males; mean age = 24.7; SD = 6.1) who each underwent a full-body illusion eight minutes in duration. Before and after the illusion, participants indicated their perceived (shifts in) self-location or “proprioceptive drift.” This was done on a measuring tape that counted 0 cm out from the participant towards the screen positioned 200 cm away from them (*behavioral measure*; Fig. 5b). Therefore, 0 cm indicated no displacement towards the virtual body (i.e., self-location is perceived inside the physical body), whereas everything else indicated illusory displacement of various gradations (i.e., 200 cm max.; what is felt and seen during the illusion is perceived as originating from the virtual body). Lastly, participants completed a 5-minute exit interview consisting of 15 statements explicitly measuring their experiences. For example *“I felt a shift from my body towards the virtual body,”* was measured on 5-point Likert scales ranging from “*1 = Strongly Disagree*” to “*5 = Strongly Agree.*” Based on their answers “total interview scores” were calculated (*psychometric measure*; for details see Supplementary Materials). The study was fully randomized, double-blind, and sham-controlled. It featured a within-subjects design that included two sessions: half of the participants received 25 minutes (1500 seconds) of anodal stimulation in Session 1 (day 1); the other half received 1500 seconds of anodal stimulation in Session 2 (day 8). On all other occasions, sham stimulation was administered. Sessions were held one week apart to avoid carryover effects. Statistics were performed in SPSS version 25.0 and reported using a 0.05 significance level.

The results confirmed that the novel FBI-paradigm had been successful. The average reported displacement towards the projected image was 69.1 cm in Session 1 and 72.4 cm in Session 2. Maximal displacement was reported on nine occasions (180–200 cm); while 1/6 of participants reported no displacement (17.1%). A first mixed ANOVA^8^ found a significant main effect for displacement in pre- and post-test scores over sessions, *F*(1,31) = 62.8, *p* < 0.001, ηp^2^ = 0.67 (behavioral measure). Likewise, a second mixed ANOVA performed over the total interview scores found a significant main effect in reported experiences over sessions, *F*(1,31) = 5.2, *p* = 0.03, ηp^2^ = 0.14 (psychometric measure). In addition, a strong positive correlation between the amount of displacement (behavioral measure) and reported experiences (psychometric measure) was shown, *r*(33) = 0.57, *p* < 0.01 (Session 1) and *r*(33) = 0.51, *p* < 0.01 (Session 2), one-tailed Bonferroni corrected. Finally, there was also a significant effect of anodal right angular gyrus stimulation: the first mixed ANOVA revealed a significant interaction in displacement scores between experimental groups, *F*(3,31) = 4.4, *p* = 0.01, ηp^2^ = 0.30. Group 1: HD-tDCS S1 “*Yes” M* = 52.1(6.7) vs. S2 “*No” M* = 48.7(7.7); Group 2: HD-tDCS S1 “*No” M* = 18(6.5) vs. S2 “*Yes” M* = 24.4(7.5), Bonferroni corrected. See (de Boer et al., 2020) for more results, including full details on the statistical analysis and study protocol.

## Future applications and broader scope

FBIs are typically simulated in a virtual environment using head-mounted displays (HMDs). However, HMDs are not easily combined with neuroscience methods that generally require free and unobstructed access to the head and skull. As a consequence of these challenges, the neural mechanisms that underpin such illusions remain understudied. To address these problems, this paper provided a step-by-step guide on how to create and integrate *3D LIVE-streaming* into a real-life FBI paradigm. This was complemented by highlighting some current practical, technical, and safety concerns of combining HMDs with noninvasive brain stimulation (NIBS) techniques. In return, we provided evidence demonstrating the effectiveness of our methods. Lastly, this section gives an overview of how our methods fit within current approaches and will end with suggestions for future applications.

Beneficial for current research, this novel FBI paradigm allows unobstructed access to the head and skull. This gives the researcher full freedom to safely record and/or manipulate the brain functions of their participants. As such, this paradigm can be combined with a range of neuroscience methods, e.g., EEG, NIRS, tDCS, TMS, (invasive) single-unit recordings, and neurostimulation.^9^ To our knowledge, our study was the first to combine brain stimulation with an FBI paradigm, which allowed us to shed light on the casual origin of disembodied experiences and neural basis of self-identification (see discussion on *minimal phenomenal selfhood* (Windt, 2010)*, (*Metzinger*,* 2013*)*). Importantly, this novel FBI-paradigm appears to be an effective way to study the neural underpinnings of out-of-body experiences: our preliminary results revealed a causal role for the right angular gyrus in self-location mediated perspective-taking ( (de Boer et al., 2020)). An interesting question at this point is how the behavioral results of this novel FBI-paradigm compare to the original results obtained using VR. Looking at the data, our results appear to neatly fit in with previous results measuring illusory self-location (see Fig. 4 in (Dieguez & Lopez, n.d.)). However, instead of comparing synchronous versus asynchronous stroking conditions as is generally done in HMD paradigms, we measured self-location (before and) after participants received anodal versus sham (baseline) stimulation and synchronous back-stroking (note: the stimulation order was random, see Section 3). Adding to the previous results, more illusory displacement (proprioceptive drift) was reported after active versus sham right angular gyrus stimulation (see Fig. 2 in (de Boer et al., 2020)). Also, the way “proprioceptive drift” was measured was slightly different. In the novel FBI paradigm, participants were asked to point out (in centimeters on a measuring tape) perceived shifts in self-location before and after the illusion (Fig. 5), whereas in previous HMD paradigms, blindfolded participants were passively displaced after the illusion and asked to walk back to their original location, and difference measures were taken (Fig. 4 in (Dieguez & Lopez, n.d.)). Nevertheless, considering the similar results, both approaches appear to be a feasible way to measure illusory displacement. Importantly, this also implies that participants can be explicitly questioned about their experiences, giving more detailed insight into them ( (Dieguez & Lopez, n.d.)). 10 In addition, this also provides an opportunity to verify the experiences and internal validity of the FBI paradigm (including proper verification of the involved neural target). Subsequently, this is the first FBI-paradigm that directly compared a variety of measures and tasks to critically look at disembodied experiences and its neural substrate; a behavioral measure (proprioceptive drift) was systematically compared to a psychometric measure (exit interview) and a control perspective-taking task (i.e., measuring the effect of stimulation in the opposite direction) with and without anodal stimulation. Also, the exit interview tested multiple facets of the illusory experience, i.e., displacement, self-identification, and sense of agency (Table S1). Most notably, sense of agency (Gallagher, 2000; Haggard & Chambon, 2012), i.e., perceiving control in one’s actions (and thoughts), has previously been left out in the examination of FBIs. Lastly, participants estimated (a) the onset time of the displacement from “*0* = *never*,” “*1* = *after a while”* to “*2* = *fairly quickly*,” (b) the onset time in minutes, including (c) the frequency of the displacement from “*0* = *never*,” “*1* = *once shortly*,” *2* = *many short times*” to “*3* = *continuously*.” In completion, the original reports were verified and contrasted to the answers participants provided to an open question describing their experience (see Supplementary Materials). This combination of measures, including the addition of HD-tDCS that enhanced the illusory experiences, provided a more comprehensive picture of (what causes) the breakdown in the unity between self and body (for details see (de Boer et al., 2020)).^11^

It is also important to highlight that the methods discussed in this paper are very different from the ones that are currently commercially available. For example, the CAVE (Cave Automatic Virtual Environment (Visbox, Inc (Illinois, USA), 2020)) systems have also been developed to overcome the limitations of using HMDs for scientific and engineering purposes. These systems use large projection panels to create life-size artificial environments or “caves” that users can move through and interact with. However, all this high-tech equipment comes at a cost and with cumbersome hardware (for a recent review (Manjrekar et al., 2014)). More importantly, CAVE systems cannot be used to create a FBI paradigm by itself. One problem is how the illusion is induced. When the FBI is not computer-simulated in VR (using an HMD), a camera setup should somehow record observers, whose images need to be fed back to them to induce the illusion. Therefore, a technique *like 3D LIVE-streaming* should be introduced into the system to recreate an illusion. Our method does this without a CAVE system. Thus, 3D LIVE-streaming is a technique that can potentially be used for a variety of purposes, not only as a projection. In principle, 3D images can be streamed to any device or screen that can display 3D images, be it large or small (e.g., a monitor, tablet, mobile phone). Another important benefit of streaming is that anything that can be captured with a camera does not need to be produced. We effectively film our participants instead of taking pictures of them and mapping that onto a 3D humanoid in an artificial environment (i.e., the proper way to simulate self-generated movement in an avatar; e.g., (Swinkels et al., n.d.) used premade videos). The latter always produces qualitatively inferior results and consumes more time and resources to produce. In addition, CAVE systems are more rigid commercial products that need sufficient lab space to be properly set up (e.g., the novel FBI paradigm requires 4×2 meters space). They make use of large, fixed screens positioned further away from the observer, which is not suitable to set up a FBI paradigm or very useful in standard clinical practice ( (Manjrekar et al., 2014)). Our setup is low-budget (costs ~ $2000^12^), high quality, and adaptable to suit the needs of different users. Importantly, it should be quite easy to set up with the guide provided under Sections 2 and 3. Finally, it is not beneficial to be dependent upon rigid and expensive commercial products with offsite tech support when the bulk of the creative development still has to be done by the researchers (note: this might be different when a lab is devoted to one purpose, e.g., as therapy for post-traumatic stress (Wout-Frank et al., 2019)). In contrast, the methods presented here could greatly benefit researchers and future clinicians that require flexible and adaptable use of tools with real-time high-quality output.

3D LIVE-streaming can potentially benefit a variety of purposes, users, and settings. Another prominent difference between this novel FBI paradigm and (most) previous ones is that participants effectively looked at themselves to evoke the illusion (see original paradigms (Ehrsson, 2007; Lenggenhager et al., 2007)). In other words, the illusion was evoked without the creation of a computer simulation. In this respect, this FBI paradigm is similar to mirror paradigms (Ramachandran & Altschuler, 2009), which, among others, have been shown to be beneficial in the recovery from phantom limb pain (for a recent review (Campo-Prieto & Rodríguez-Fuentes, 2020)). Oftentimes, simple techniques are more ecologically valid and generate the best results. Therefore, like mirror paradigms, the current methods could offer a simple and effective way to create realistic, self-generated movement under both experimental and therapeutic conditions (note: a high frame rate adds to this realism, e.g., 60 fps). In the former case, for example, this enables one to investigate whether and how full-body illusions can extend to conditions with active control in movements, such as to an avatar in a virtual world. Can the brain adapt or learn to take on another’s bodily perspective, much like it can incorporate prostheses and external body parts ( (Giummarra et al., 2008)*;* for preliminary data see (Swinkels et al., n.d.), (Banakou et al., 2013), (Kondo et al., 2020))? Furthermore, future rehabilitation programs may benefit from the inclusion of experimental tasks and/or neurostimulation to aid recovery from nervous system injury (e.g., spinal cord injury, brain damage, limb loss) or to alleviate psychosis symptoms (e.g., (Frith, 2005; Pynn & DeSouza, 2013)). An important part of our research is investigating the potential prominent role of the right angular gyrus (and involved networks) in self-identification, and, particularly, in distinguishing self from other signals. This is based on the hypothesis that the self (i.e., internal vs. external signals) is coded with reference to its location in space (de Boer et al., 2020). For example, self-produced signals involve the early mobilization of predictions in the brain (e.g., efference copies, corollary discharge (Frith, 2005; Pynn & DeSouza, 2013)). Such predictions may enable the brain to discriminate self-produced signals from environmental stimulation at an early stage and pre-recruit relevant attention networks (see model in Fig. 1 (de Boer et al., 2020)). One of the most challenging issues in neurorehabilitation is establishing accurate relationships between sensory processing and internal representations of the body (Perruchoud et al., 2016). Our proposed model offers new insight into these processes, and can inform future rehabilitation programs to guide stimulation targets aiming to restore proper sensory processing and self–other discrimination (i.e., self-identification; see Note 9). More specifically, such targeted stimulation could result in a better balance of sensorimotor transformations and internal body representations (i.e., neurons in the posterior parietal cortex convert various representations of space into a common, egocentric frame of reference (Blanke, 2012; Giummarra et al., 2008)). Alternatively, the methods presented also have potential future clinical and commercial benefit. As was highlighted in this paper, 3D LIVE-streaming is not GPU-intensive and can easily be applied to any device or screen that can display 3D images (e.g., TV, tablet, mobile phone). Importantly, 3D LIVE-streaming could be used to enhance future clinical observations or educational tools, or potentially guide medical interventions with real-time high-quality 3D images. Finally, 3D LIVE-streaming has the potential to set a new standard for immersive online gaming as well as augmenting online and mobile experiences (e.g., video chat, social sharing/events).

## Supplementary Information

ESM 1 (DOCX 1.76 mb)

## Footnotes

1 There is currently scientific consensus that body ownership and self-identification are interchangeable. Our new findings and model challenge this view (de Boer et al., 2020).

2 Windows OS does not currently support two or more USB ports as streaming feeds.

3 For streaming long periods on end we recommend using cameras with USB adapters. Standard rechargeable batteries deplete quickly (e.g., 20–30 min). A buffer may build up in Bino 3D Player that can cause image delays and result in a software crash.

4 Cassani et al. (2020) state: “It is important to note that the 3 articles, [34, 35] and [36], that used head-based VR systems, relied on VR devices that are 10 years or older, thus they may not be appropriate for realistic VR experiences” (Cassani et al., n.d.).

5 A magnetometer measures head-orientation by picking up surrounding magnetic fields (e.g., the Earth’s magnetic North), and can even react to a random magnetized object lying on the table; a gyroscope oscillates at a high frequency to measure head-rotation relative to itself, which can be affected by other vibrations and alternating currents (LaValle, 2020).

6 Our participants were videotaped instead of taking pictures of them and mapping that to a 3D humanoid in an artificial environment (e.g., important when participants can move freely). The latter produces qualitatively inferior results and costs more time and resources to produce.

7 Since participants were illuminated from the back, it was important to make sure that the spotlights did not shine directly into the 3D goggles they were wearing. Premade cotton cloths were attached to the goggles’ temples to avoid this from happening (Fig. 3).

8 The study design was mixed between-subjects and within-subjects (repeated measures): it included two tasks (full-body illusion; perspective taking not discussed) and two types of stimulation (anodal vs. sham). Full randomization resulted in four experimental groups with AB/BA stimulation protocols (de Boer et al., 2020).

9 Because participants are videotaped from behind, this particular setup is not suitable to combine with MEG or fMRI/PET.

10 Participants with high susceptibility traits were screened out in our study (de Boer et al., 2020).

11 Future studies can extend this FBI paradigm to dissociate the role of the right angular gyrus (exogenous or stimulus control) from prefrontal cortex function (endogenous or cognitive control) in self-location (Fig. 1 (de Boer et al., 2020)). This will provide more conclusive evidence for the critical involvement of attention networks in self-identification (i.e., self–other discrimination).

12 The setup included two budget action cameras (2K, 60 fps; $70 each), a midrange full-HD projector ($500), a top-range laptop (i7, 16 GB; $1000–1500), and capture cards (4K, 60 fps input, 1080 p 60 fps output; $60 each, 2018).

## Declarations

### Data and/or Code availability

The data and materials of the experiment reported in Section 3 are available at 10.17605/OSF.IO/B9TKU. The experiment was not preregistered.
