# Supplementary material for: LIVE-streaming 3D images: A neuroscience approach to full-body illusions
Source: Behav Res Methods. 2021 Sep 28;54(3):1346–57. doi: 10.3758/s13428-021-01659-6 (PMC9170653; doi:10.3758/s13428-021-01659-6)
Supplement: Supplementary file 1 — (DOCX 1.76 mb) [file 13428_2021_1659_MOESM1_ESM.docx]

Supplementary Materials for

*LIVE Streaming 3D Images: A Neuroscience Approach to Full-body Illusions*

D.M.L. de Boer^1,2^*, F. Namdar^,3^, M. Lambers^4^, A. Cleeremans^5^.

Correspondence to: [debbie.boer@hdr.qut.edu.au](mailto:debbie.boer@hdr.qut.edu.au)

**This file includes:**

Table S1

Fig. S1-2

Data S1

**Other Supplementary Materials for this manuscript include the following:**

Data S2 (OSF)


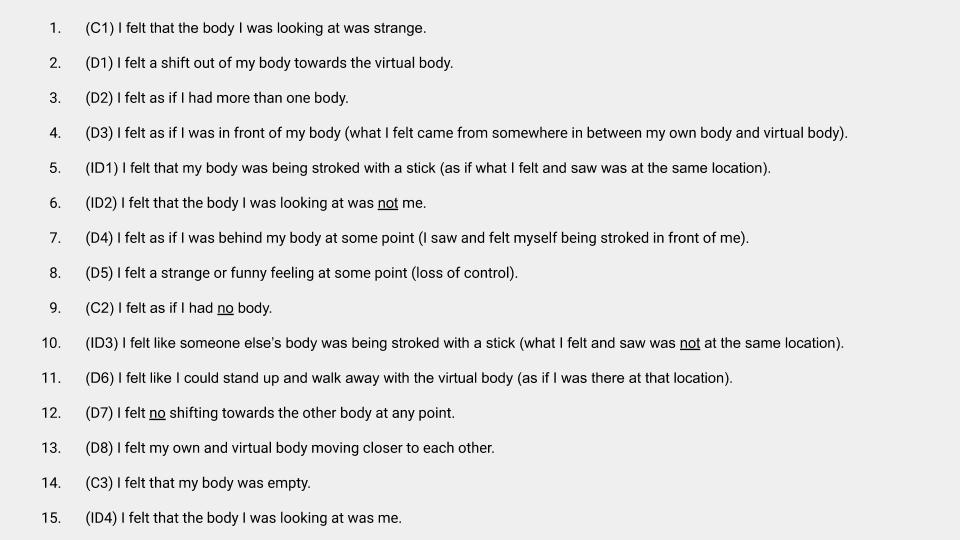


**Table S1. Full-body Illusion (FBI) Exit Interview (psychometric measure).**

Listed items were answered and scored on 5-point Likert scales ranging from ‘*5 = Strongly Agree*,’ ‘*4 = Agree*,’ ‘*3 = Neither agree or disagree*,’ ‘*2 = Disagree*’ to ‘*1 = Strongly Disagree’* (negatively phrased items reverse-coded); Item Codes: D1-8 Displacement; ID1-4 Self-Identification; C1-3 Control (C1 measured ‘General Disposition’ to illusion between sessions); D5 & D6 measured changes in perceived control, i.e., ‘Sense of Agency’ (answers were verified with response to open question: ‘*Please describe the displacement in a few words*’, see **Fig. S1**).

**Fig. S1. 3D LIVE Streaming using Capture Cards (August 2018)**


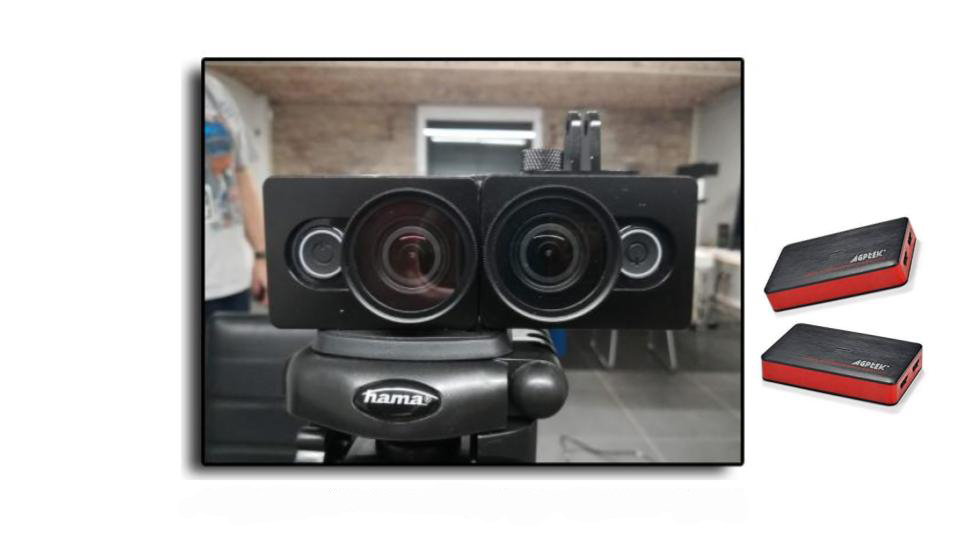


LIVE streaming stereoscopic 3D images in full HD at 60 frames per second using two budget Action Cameras (brand: Xiaomi Yi, 2K resolution), two USB 3.0 Capture Cards (brand: AGPtek, 4K resolution, 60 fps input; 1080P, 60 fps output) and Bino 3D Player-software (Lambers, 2012).


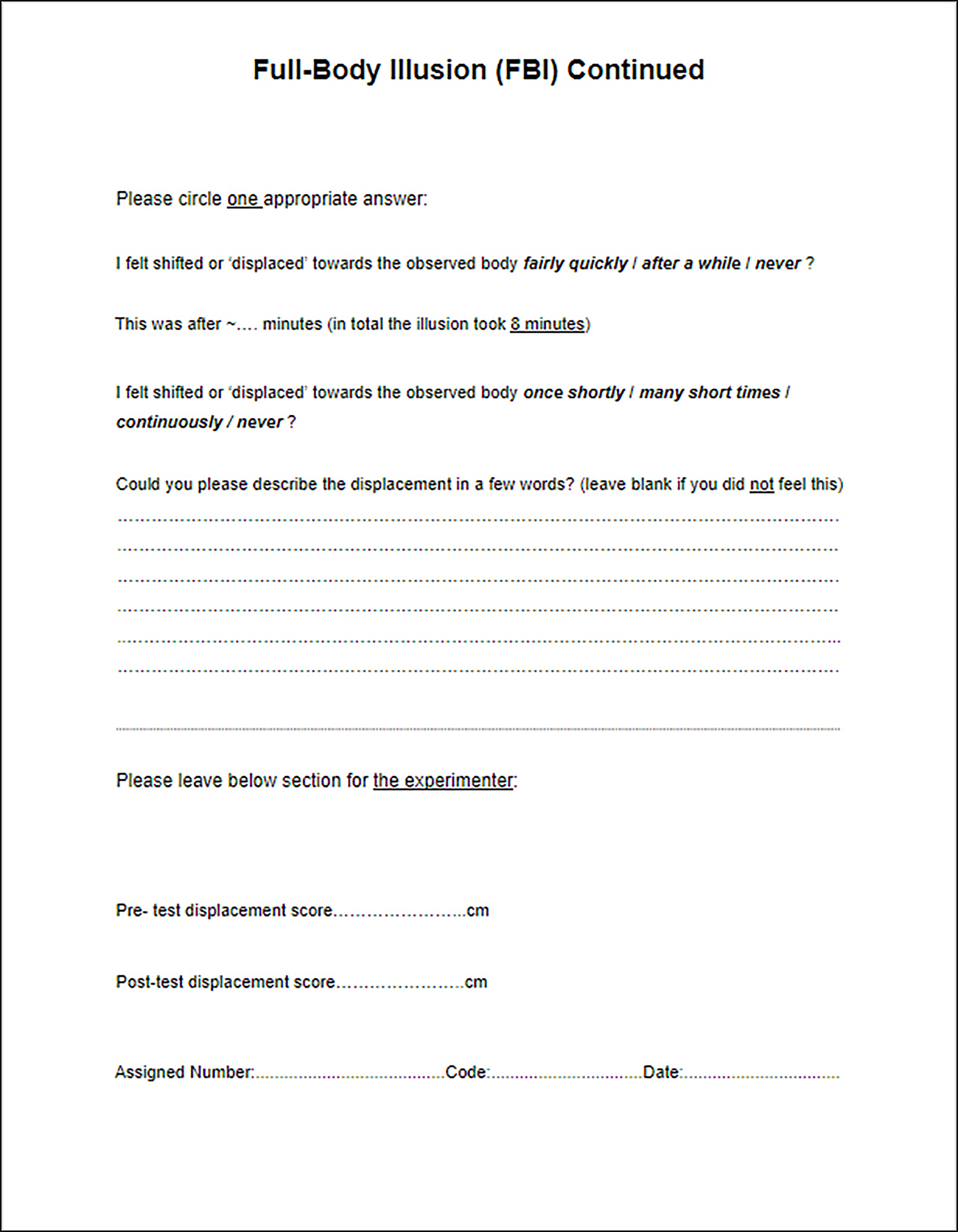


**Fig. S2. Proprioceptive drift and other measures of ‘Displacement’**

Above: Participants estimated (i) the onset time of the displacement from *‘0 = never,’ ‘1 = after a while’* to *‘2 = fairly quickly,’* (ii) the onset time in minutes, and (iii) the frequency of the displacement from *‘0 = never,’ ‘1 = once shortly,’ 2 = many short times’* to *‘3 = continuously.’* Answers to the FBI Exit Interview (psychometric measure; Table S1) were verified with the answers participants provided to (iv) an open question describing their experience.

Below: Before (pre-test) and after the illusion (post-test) participants pointed out in centimeters (perceived shifts in) self-location, i.e., proprioceptive drift (behavioural measure; Fig. 5b).

**Data S1. Full-body Illusion (FBI) Exit Interview**

The FBI Exit Interview consisted of 15 items (incl. 3 control items) that were answered and scored on 5-point Likert scales. There were eight *‘Displacement Items,’* two of them measured sense-of-agency (i.e., *‘Agency low’* & *‘Agency high’*), and four *‘Self-Identification Items,’* see Table S1. Negatively phrased questions were reverse coded and did not indicate inconsistencies in answers. Subsequently, a high score on each item (excl. control items) represented a more pronounced full-body illusion. Descriptive statistics pooled over the sessions confirmed that all items (except control items 9 & 14) had a mean score > 3 points (Item 11 = 3; measuring maximal displacement) and SD > 1 point (except Item 15). As expected, the displacement items (Items 2, 3, 4, 7, 8, 11, 12 & 13) were strongly correlated, *r*(33)  > 0.5, *p* = 0.01; while other items were not correlated, *r*  = 0 one-tailed Bonferroni corrected. Inter-item correlations measured with Cronbach’s α: 0.89 Session 1; 0.87 Session 2 (displacement items); 0.81 Session 1; 0.80 Session 2 (15 items excl. control items 1, 9 & 14). *“Total Exit Interview Scores”* were calculated excluding the control items.

Data S2. (OSF) <https://osf.io/b9tku/>
